# Volume Measurements of the Knee Articular Cartilage and Epiphyseal Bone for Evaluating the Structural Characteristics of the Discoid Lateral Meniscus

**DOI:** 10.7759/cureus.98475

**Published:** 2025-12-04

**Authors:** Keita Nagawa, Yuki Hara, Shinji Kakemoto, Hirokazu Shimizu, Saki Tsuchihashi, Naoki Sugita, Kaiji Inoue, Eito Kozawa

**Affiliations:** 1 Radiology, Saitama Medical University Hospital, Saitama, JPN; 2 Radiology, Japanese Red Cross Ogawa Hospital, Saitama, JPN; 3 Orthopedics, Saitama Medical University Hospital, Saitama, JPN

**Keywords:** discoid lateral meniscus, epiphyseal bone, knee articular cartilage, three-dimensional magnetic resonance image, volume analysis

## Abstract

Introduction

Previous studies have identified morphological features in the bone and cartilage of knees with discoid lateral meniscus (DLM), but these investigations often depended on simple geometric variables like angles and linear dimensions. Three-dimensional (3D) bone and articular cartilage models can be used to obtain the volumes of knee structures. This study aimed to evaluate the volume of articular cartilage and epiphyseal bone in non-pathological knees and those with DLM and to assess their structural differences.

Materials and methods

This study included 19 magnetic resonance imaging (MRI) scans of knees in 16 patients with DLM and 31 MRI scans of non-pathological knees in 30 patients. Knee articular cartilage and epiphyseal bone segmentation were performed to obtain 3D reconstructed models. Subsequently, the cartilage and bone models were divided into five compartments for the femur (two-row (anterior and posterior) sections for the lateral and medial condyles and one section for the intercondylar region) and six compartments for the tibia (using a three-column (medial, intercondylar, and lateral) × two-row (anterior and posterior) grid), and the volumes of each compartment were calculated.

Results

The DLM knees had reduced volume in the lateral part of the articular cartilage and epiphyseal bone than those in the control group. Statistically significant differences between the two groups were observed in the volumes of the lateral tibial cartilage and lateral femoral epiphyseal bone.

Conclusions

Our study showed that the volume measurements of the knee articular cartilage and epiphyseal bone could facilitate the understanding of the structural differences between knees with DLM and non-pathological knees. The lateral compartment of the articular cartilage and bone is smaller in knees with DLM, and may be distinguishable from non-pathological knees.

## Introduction

The discoid lateral meniscus (DLM) is a common anatomic variant of the knee, with a wide, hypertrophic, and discoid-shaped meniscus. DLM typically presents in young populations, with a greater incidence in the Asian population than in other ethnic groups [1,2]. Because DLM is congenital, its morphological and structural characteristics differ from those of a normal meniscus, potentially leading to degeneration and tears [3-5]. Conservative management is preferred when the patient is asymptomatic and the meniscal rim is maintained. If this approach is unsuccessful, meniscal repair with partial or total resection can be performed. The treatment strategy differs from that for a normal meniscus [6,7].

The major radiological findings of DLM include widening of the lateral joint line, cupping of the lateral tibial plateau, squaring of the lateral femoral condyle, widening of the tibial eminence, elevation of the fibular head, and condylar cutoff sign [8-12]. These findings are essentially based on plain radiographs, which are useful for initial screening; however, the results for each parameter are less reproducible and subject to interobserver bias.

Magnetic resonance imaging (MRI) is also an important diagnostic modality for DLM [13,14]. Although MRI can detect abnormalities in both the bone and surrounding soft tissue, determining the presence of a DLM can be difficult in cases such as meniscal tears because the shape of the DLM can be mistaken for deformed bucket-handle and inverted flap tears [15]. Therefore, having prior knowledge of other specific indicators of DLM would be beneficial, and several researchers have focused on bone morphology. Hypoplasia of the posterior lateral femoral condyle has been reported to be an indicator of DLM [16]. More recently, a three-dimensional (3D) MRI-based evaluation of bone morphology has further demonstrated hypoplasia of the lateral femoral condyle and posterior lateral tibial plateau in patients with DLM [17]. Morphological changes in the articular cartilage associated with DLM have also been reported. The thickness of the articular cartilage is related to meniscus coverage, which acts as a load distributor and shock absorber [18]. Moreover, the articular cartilage of the tibia is thicker in the area not covered by the meniscus [18]. A recent study demonstrated that the articular cartilage of the lateral tibial plateau was thinner in patients with DLM than in those of controls, which might be due to excessive coverage of the DLM [19].

Thus, the morphological features of bone and cartilage may be useful findings suggestive of DLM. However, the methods used in previous studies were based on a limited set of conventional geometric variables, such as the angle and thickness [17,19]. Alternatively, 3D models of bones and articular cartilage can be utilized to obtain the volumes of these structures, either whole or in sections, which can be useful in terms of reliability and reproducibility. At our institution, 3D T2*-weighted fast-field echo (T2* FFE) is routinely used in knee studies. Although this sequence is primarily intended for the high-resolution evaluation of cartilage, other tissues, including the bone, are also clearly visualized. The 3D T2* FFE method has the advantage of using volumetric data for 3D segmentation and volumetric analysis.

To the best of our knowledge, no studies have evaluated the volume of articular cartilage and subchondral bone in relation to the DLM. Therefore, in this study, we aimed to calculate the volume of cartilage and epiphyseal bone in knees with DLM and in non-pathological knees, and to evaluate the structural differences between the two.

## Materials and methods

Subjects and image acquisitions

This study was approved by the Research Ethics Committee of Saitama Medical University Hospital, Saitama, Japan (approval number 2023-046). The requirement for informed consent to participate was waived by the Institutional Review Board of Saitama Medical University because of the study’s retrospective design. All experiments were performed in accordance with relevant guidelines and regulations.

After receiving institutional review board approval, we reviewed the records of patients aged <30 years who underwent knee MRI between January 2017 and September 2021, requested by the Department of Orthopedics in our hospital. All patients diagnosed with DLM at our institution were included in this study. DLM diagnosis was based on the criteria of Watanabe et al., requiring a meniscus width covering more than 80% of the tibial plateau on coronal MRI views [2,5], and confirmed by an experienced musculoskeletal radiologist. While all patients initially underwent MRI before diagnosis, those with knee pain due to other etiologies such as fracture, arthritis, chondrosis, osseous stress response, or high-grade ligament sprains were excluded. Age- and sex-matched controls were also identified, defined as those with non-pathological knee MRI findings and no clinical knee abnormalities. Controls were defined as those with knee MRI findings showing no evidence of meniscal tears, ligament injuries, osteoarthritis, or other significant pathology, and who had no clinical history of knee pain or injury at the time of the MRI. Subjects with fractures, arthritis, chondrosis, osseous stress responses, or high-grade ligament sprains were excluded. Thus, 19 MRI scans from 16 patients with DLM knees (sex ratio 7/9 (male/female); age 20.5 ± 5.3 years (mean ± standard deviation); side ratio 7/12 (right/left), including both sides of the knees in three patients) and 31 MRI data from 30 patients with non-pathological femurs (sex ratio 13/18 (male/female); age 20.5 ± 3.9 years (mean ± standard deviation); side ratio 16/15 (right/left), including both sides of the femurs in one patient) were examined in the study.

All MRI scans were performed using a 3.0-T system (Ingenia Elition, Philips Healthcare, Best, The Netherlands) with a vendor-specific 16-channel knee coil. In addition to routine knee imaging protocols, including axial, sagittal, and coronal proton density imaging, the 3D T2* FFE sequence was performed in all patients. The implementation protocol for 3D T2* FFE was as follows: repetition time, 15 ms; echo time, 5 ms; flip angle, 30°; slice thickness, 1.5 mm; field of view, 15.0 × 17.1 cm; voxel size, 0.5 × 0.5 × 1.5 mm; in-plane resolution, 320 × 224.

Measurement of the knee articular cartilage and epiphyseal bone volume

**Figure 1:**
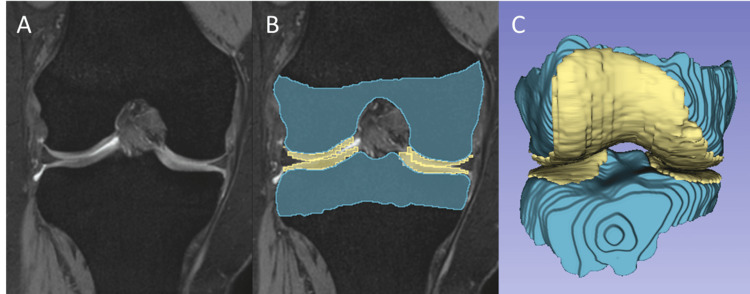
The 3D model reconstruction of the knee articular cartilage and epiphyseal bone (A) A coronal reconstructed image is presented to illustrate the 3D segmentation process. (B) The areas of the knee articular cartilage and epiphyseal bone were delineated on each slice using semi-automatic segmentation tools. The area of the epiphyseal bone was delineated by the epiphyseal line, which was visible on sagittal and coronal views. (C) From the delineated contour, the 3D model of each articular cartilage and epiphyseal bone was reconstructed.

To measure the volumes of the knee articular cartilage and epiphyseal bone, 3D segmentation was first performed using open-source software (ITK-SNAP version 3.8.0). After the MRI scans were loaded into ITK-SNAP in the Digital Imaging and Communications in Medicine (DICOM) format, the areas of the knee articular cartilage and epiphyseal bone were delineated on every slice using semi-automatic segmentation tools (Figure 1). Segmentation was performed by two trained radiologists with 9 and 10 years of experience in musculoskeletal MRI segmentation. To assess inter-observer reliability, 10 randomly selected cases were segmented by both operators, and the intraclass correlation coefficient (ICC) was calculated for the volumetric measurements. The ICC values were excellent, ranging from 0.92 to 0.96 for all compartments. Any discrepancies in segmentation were resolved by consensus. The epiphyseal bone area was delineated proximally using the epiphyseal line, which was visible in sagittal and coronal views. From the delineated contour, a 3D model of each articular cartilage and epiphyseal bone was reconstructed and saved as a standard 3D model in the NIFTI file format (*.nii.gz). The 3D surface models were subsequently loaded into an open-source software package (3D Slicer version 5.0.3) for further analysis.

**Figure 2:**
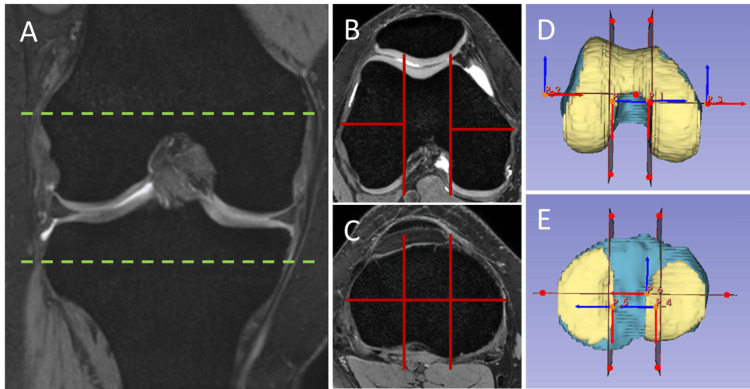
Sectional volume measurements of knee articular cartilage and epiphyseal bone As annotated on (A) a cross-referenced coronal reconstructed image, axial images through the (B) femoral and (C) tibial condyles are shown. To ensure consistent and detailed volume measurements, sectional volume measurements of knee articular cartilage and epiphyseal bone were performed. (D) For the femur, the cartilage and bone models were divided into a total of five compartments (two-row (divided into anterior and posterior parts) sections for the lateral and medial condyles, and one section for the intercondylar part). (E) For the tibia, the bone model was divided into a total of six compartments (using a three-column (divided into medial, intercondylar, and lateral parts) by two-row (divided into anterior and posterior parts) grid), and both the lateral and medial cartilage models were divided into anterior and posterior parts.

To ensure consistent and detailed volume measurements, we performed sectional volume measurements of the knee articular cartilage and epiphyseal bones. The cartilage and bone models were divided into five compartments for the femur (two-row (divided into anterior and posterior parts) sections for the lateral and medial condyles and one section for the intercondylar part) and six compartments for the tibia (using a three-column (divided into medial, intercondylar, and lateral parts) by two-row (divided into anterior and posterior parts) grid), as shown in Figure 2. The cartilage of the tibia was divided into four compartments (two-row (divided into anterior and posterior) sections for the lateral and medial compartments). The volume of cartilage and bone for each section was measured and stored in series, and the Student’s t-test was used to compare continuous variables between the DLM and control groups. All procedures were conducted using open-source software (3D Slicer 5.0.3). Statistical analyses were performed using the open-source software package (Python scikit-learn 0.22.1). Statistical significance was set at p < 0.05.

## Results

A comparison of knee articular cartilage volumes between the DLM and control groups is shown in Tables 1-2. Regarding the femoral cartilage, no statistically significant difference was found between the two groups. Although the difference was not statistically significant, the mean volume of the lateral femoral cartilage was smaller in the DLM group than in the control group. The mean volume of the lateral tibial cartilage was significantly smaller in the DLM group than in the control group; however, no significant differences were observed in the medial and intercondylar compartments.

**Table 1:** A comparison of the femoral cartilage volume between DLM and control groups Data are presented as mean ± standard deviation unless otherwise indicated. p-values indicate a statistically significant difference between groups (p < 0.05). Cohen’s d is a standardized effect size for measuring the difference between the groups (0.2, 0.5, and 0.8 are considered as small, medium, and large effect sizes, respectively). DLM, discoid lateral meniscus

Compartment	DLM	Control	p-value	Cohen’s d
Lateral compartment (1 + 2)	4.33 ± 1.17	4.97 ± 1.61	0.071	0.432
Anterior compartment (1)	1.98 ± 0.52	2.31 ± 0.79	0.060	0.459
Posterior compartment (2)	2.35 ± 0.71	2.66 ± 0.88	0.099	0.378
Intercondylar compartment (3)	2.82 ± 1.12	2.72 ± 0.75	0.347	0.115
Medial compartment (4 + 5)	3.43 ± 0.91	3.53 ± 0.92	0.362	0.103
Anterior compartment (4)	0.99 ± 0.31	1.01 ± 0.32	0.407	0.069
Posterior compartment (5)	2.45 ± 0.64	2.52 ± 0.65	0.350	0.112

**Table 2:** A comparison of the tibial cartilage volume between DLM and control groups Data are presented as mean ± standard deviation unless otherwise indicated. p-values indicate a statistically significant difference between groups (p < 0.05). Cohen’s d is a standardized effect size for measuring the difference between the groups (0.2, 0.5, and 0.8 are considered as small, medium, and large effect sizes, respectively). DLM, discoid lateral meniscus

Compartment	DLM	Control	p-value	Cohen’s d
Lateral compartment (1 + 2)	1.77 ± 0.74	2.35 ± 0.68	0.004	0.814
Anterior compartment (1)	0.53 ± 0.32	0.78 ± 0.26	0.002	0.872
Posterior compartment (2)	1.24 ± 0.55	1.57 ± 0.48	0.016	0.638
Medial compartment (5 + 6)	1.84 ± 0.69	1.91 ± 0.58	0.356	0.108
Anterior compartment (5)	0.80 ± 0.28	0.83 ± 0.30	0.369	0.098
Posterior compartment (6)	1.04 ± 0.44	1.08 ± 0.33	0.363	0.102

Comparisons of the knee epiphyseal bone volumes between the DLM and control groups are shown in Tables 3-4. Regarding the femoral epiphyseal bone, the mean volume of the lateral compartment, particularly the posterior compartment, was significantly smaller in the DLM group than in the control group; however, no significant difference was observed in the medial and intercondylar compartments. No statistically significant difference was found in the tibial epiphyseal bone between the two groups.

**Table 3:** A comparison of the femoral epiphyseal bone volume between DLM and control groups Data are presented as mean ± standard deviation unless otherwise indicated. p-values indicate a statistically significant difference between groups (p < 0.05). Cohen’s d is a standardized effect size for measuring the difference between the groups (0.2, 0.5, and 0.8 are considered as small, medium, and large effect sizes, respectively). DLM, discoid lateral meniscus

Compartment	DLM	Control	p-value	Cohen’s d
Lateral compartment (1 + 2)	27.26 ± 6.22	32.29 ± 8.46	0.016	0.640
Anterior compartment (1)	12.63 ± 2.49	14.46 ± 3.81	0.036	0.531
Posterior compartment (2)	14.63 ± 3.87	17.83 ± 4.80	0.010	0.700
Intercondylar compartment (3)	16.77 ± 4.68	14.74 ± 4.30	0.064	0.448
Medial compartment (4 + 5)	30.99 ± 7.23	32.56 ± 8.18	0.251	0.196
Anterior compartment (4)	11.44 ± 2.51	11.98 ± 3.04	0.260	0.188
Posterior compartment (5)	19.56 ± 4.84	20.58 ± 5.34	0.253	0.194

**Table 4:** A comparison of the tibial epiphyseal bone volume between DLM and control groups Data are presented as mean ± standard deviation unless otherwise indicated. p-values indicate a statistically significant difference between groups (p < 0.05). Cohen’s d is a standardized effect size for measuring the difference between the groups (0.2, 0.5, and 0.8 are considered as small, medium, and large effect sizes, respectively). DLM, discoid lateral meniscus

Compartment	DLM	Control	p-value	Cohen’s d
Lateral compartment (1 + 2)	14.6 ± 3.9	15.8 ± 4.3	0.168	0.282
Anterior compartment (1)	8.68 ± 2.30	9.06 ± 2.61	0.306	0.148
Posterior compartment (2)	5.89 ± 1.73	6.70 ± 1.83	0.066	0.444
Intercondylar compartment (3 + 4)	18.47 ± 5.17	19.13 ± 4.83	0.327	0.131
Anterior compartment (3)	10.90 ± 3.23	11.28 ± 2.82	0.336	0.123
Posterior compartment (4)	7.56 ± 2.06	7.85 ± 2.11	0.322	0.135
Medial compartment (5 + 6)	11.79 ± 3.37	12.35 ± 3.06	0.278	0.172
Anterior compartment (5)	5.16 ± 1.50	5.49 ± 1.29	0.209	0.237
Posterior compartment (6)	6.64 ± 1.91	6.86 ± 1.86	0.343	0.118

## Discussion

In this study, we measured the volumes of cartilage and epiphyseal bone in knees with DLM and in non-pathological knees to evaluate the structural differences between the two. Our results showed that patients with DLM had significantly lower volumes of lateral tibial cartilage and lateral femoral epiphyseal bone (p < 0.05). To the best of our knowledge, no studies have evaluated the differences in articular cartilage and subchondral bone volumes between patients with and without DLM. Previous studies have examined the morphological changes in the articular cartilage and bone associated with DLM. Regarding bone morphology, several radiographical findings in the lateral compartment of the knee joint have been recognized as important diagnostic indicators [8-12]; however, there might be uncertainties associated with two-dimensional (2D) evaluation and inter-reader differences. In contrast, 3D evaluation of bone morphology could be valuable for an accurate understanding of the structural differences, and several approaches have been explored to date [16,17]. Regarding articular cartilage, previous studies have primarily measured cartilage thickness in relation to DLM, as the meniscus is thought to act as a load absorber and its coverage correlates with cartilage thickness [18,19]. However, the measurement approaches used in previous studies were confined to conventional geometric variables. Considering the utility of 3D geometric evaluation, the volumetric approach used in this analysis is considered suitable for accurate and reliable structural assessments.

Our study showed that the cartilage volume of the lateral tibial plateau was significantly smaller in the DLM group compared to the control group. A previous study by Brutico et al. [19] demonstrated that patients with DLM had thin articular cartilage in the lateral tibial plateau. Despite differences in evaluation methods (i.e., 2D-based versus 3D-based approaches), our results were consistent with theirs in that the thinner cartilage had reduced volume in patients with DLM compared to that in controls. Assessment of cartilage thickness is controversial, as it may induce positional bias [18,19]. Normal cartilage thickness varies depending on the measurement location and is typically related to the cartilage-to-cartilage contact; the maximal difference between zones with and without cartilage-to-cartilage contact was reported to be 50% on the lateral side of the tibial plateau [18]. Regarding the DLM, a prior study examined cartilage thickness and found no significant difference between the DLM and control groups; however, their measurement was based on the following limitations: at the midpoint of the lateral compartment in the coronal plane and at the midpoint of the DLM in the sagittal plane [20]. To reduce such positional biases, Brutico et al. [19] considered six-zone measurement of the lateral tibial cartilage thickness and found that the average cartilage thickness was significantly greater in each zone in the non-DLM group than in the DLM group. This implies that the lateral tibial cartilage volume could be uniformly smaller in any zone in the DLM group, which was also confirmed in our analysis.

In our study, no significant difference was observed between the DLM and control groups in terms of the cartilage volume of the lateral femoral condyle. Brutico et al. [19] examined the thickness of the lateral femoral cartilage and found no statistically significant differences between the DLM and non-DLM groups. In the present study, although not statistically significant, the mean volume of the lateral femoral cartilage was smaller in the DLM group than that in the control group. In previous studies evaluating bone morphology, hypoplasia of the lateral femoral condyle was observed in patients with DLM [17], which was also observed in the present study. As cartilage covers the surface of the subchondral bone, we hypothesized that cartilage volume is linked to subchondral bone development, whereas the cartilage thickness itself appears to be unaffected by the underlying bone.

In the present study, we showed that the epiphyseal bone volume of the lateral femoral condyle was smaller in the DLM group than in the control group. Previous studies have reported shape changes in the femoral bone associated with DLM. Xu et al. [16] suggested hypoplasia of the posterior lateral femoral condyle in patients with DLM, as determined by measuring the posterior lateral and posterior medial condylar angles on knee MRI. Similar attempts have been made to demonstrate hypoplasia of the femoral condyle using posterior lateral and posterior medial condylar angles [17,21]. Recently, Kinoshita et al. [17] reported that the lowest point of the lateral femoral condyle was more lateral in the DLM group than in the control group, suggesting hypoplasia of the lateral femoral condyle. DLM may cause excessive stress on the lateral femoral condyle and affect the ossification of the secondary epiphyseal nucleus [22]. Furthermore, osteochondritis dissecans of the lateral femoral condyle may develop in patients with DLM, and its pathogenesis is thought to be associated with the type and stress of DLM [23]. The mechanical stress in the lateral femoral condyle differs between patients with and without DLM; therefore, the formation of the epiphyseal bone could be affected - possibly reduced because of stress - in patients with DLM. Epiphyseal hypoplasia in the lateral femoral condyle was confirmed in our analysis, and, to the best of our knowledge, this is the first study to investigate this using volumetric analysis. In particular, this study showed that the posterior part of the lateral femoral condyle was small in DLM, which was also consistent with previous results [16,17]. It has been hypothesized that loading stress is more likely to be applied to the posterior rather than the anterior part of the femoral condyle (such as during flexion), resulting in decreased posterior epiphyseal bone formation.

Bone changes in the tibial region are reportedly associated with DLM. Widening of the lateral joint space and cupping of the lateral tibial plateau, which is normally flat or slightly convex, are well-established radiographic findings used in screening for DLM [8], and these have been suggested to originate from hypoplasia of the lateral tibial plateau. Kinoshita et al. examined the anterior and posterior obliquities of the lateral tibial plateau and demonstrated that the posterior obliquity of the lateral tibial plateau was greater in the DLM group than that in the control group, although there was no significant difference in the anterior obliquity of the lateral tibial plateau between the two groups [17]. Moreover, the joint-line obliquity causes excessive stress on the articular cartilage, leading to cartilage damage or meniscal tears [24,25]. The obliquity of the lateral tibial plateau can also be attributed to its hypoplasia [17]. In this study, although the difference was not statistically significant, the mean epiphyseal bone volume of the lateral tibial plateau, particularly the posterior part, was smaller in the DLM group than that in the control group. These results are consistent with the previous findings described above, including the posterior-dominant bony changes in the lateral tibial plateau.

Hypoplasia of the tibial eminence morphology in patients with DLM has been previously reported [11,26]. Higher tibial eminence width and tibial eminence width ratio values were found in patients with DLM than in controls [11,26]. One possible explanation for the higher tibial eminence width and tibial eminence width ratio values is that the thickened DLM impacts tibial eminence morphology, leading to its hypoplasia [26]. However, in the present study, volumetric evaluation of the intercondylar tibial epiphyseal bone did not reveal significant differences between the DLM and control groups. In our analysis, the tibial plateau was divided into the medial, intercondylar, and lateral parts using an evenly spaced three-column grid, not considering tibial eminence width or meniscal size. Therefore, geometrical differences of the tibial prominence in DLM may not be reflected in volume changes.

The observed hypoplasia of the lateral femoral condyle in DLM may represent a combination of congenital traits and developmental adaptations to altered mechanical loading. The presence of DLM during growth could influence epiphyseal ossification patterns by altering stress distribution across the knee joint. While our study did not directly assess joint-line obliquity, future research could investigate the relationship between volumetric measurements, joint-line obliquity, and clinical outcomes in patients with DLM. We acknowledge that volume changes alone cannot fully explain the pathology related to symptomatic DLM, and that a comprehensive understanding requires consideration of biomechanical factors and clinical data.

This study had some limitations. First, the sample size was small; therefore, sufficient statistical evaluation could not be performed. A post-hoc power analysis, calculated based on the observed effect size for the lateral tibial cartilage volume (Cohen's d = 0.81), revealed a statistical power of 0.72, indicating a moderate probability of detecting a true effect. We acknowledge that the relatively small sample size may limit the generalizability of our findings and increase the risk of Type II errors (false negatives). Furthermore, the control group was retrospectively identified as patients with normal knee MRI findings and no clinically abnormal knees. We acknowledge the potential for selection bias due to the retrospective selection of controls and the possibility that subtle subclinical findings or biomechanical differences could influence volumetric measurements. For proper evaluation, control subjects should be selected from healthy volunteers, and a prospective design is needed. Third, while volumetric analysis provides valuable information, it does not fully characterize the morphological variations in cartilage and bone. Future studies incorporating cartilage thickness maps, curvature analysis, and statistical shape modeling would provide a more comprehensive understanding of the structural differences associated with DLM. At the same time, while the cross-sectional volume measurements employed in this analysis are desirable for site-specific and reproducible evaluation, our approach is essentially non-automated; therefore, an automated approach for measuring these volumes should be established. Finally, the absence of clinical correlation, including symptom severity, tear pattern classification, and surgical correlation, is a limitation of our study. Future research should investigate the relationship between volumetric parameters, clinical symptoms, and treatment outcomes to determine whether volumetric measurements have prognostic value in patients with DLM.

## Conclusions

The volumes of articular cartilage and epiphyseal bone in the DLM and non-pathological knees were obtained in this study. Our sectional volume measurement revealed that patients with DLM had reduced volume in the lateral part of the knee articular cartilage and epiphyseal bone compared to that in the control group. Statistically significant differences between the two groups were observed in the volumes of the lateral tibial cartilage and lateral femoral epiphyseal bone. Further studies are needed to confirm the validity of our results and to elucidate the disease mechanism of DLM.
